# Ecological Mechanisms Underlying the Effects of Forest–Medicinal Plant Agroforestry Systems on Soil Quality and the Understory Environment

**DOI:** 10.3390/plants15192908

**Published:** 2026-09-23

**Authors:** Yuke Shi, Zihao Ye, Lingling Yan, Jiasen Wu

**Affiliations:** 1 State Key Laboratory for Development and Utilization of Forest Food Resources, Zhejiang A&F University, Hangzhou 311300, China; yukiiiishi@stu.zafu.edu.cn (Y.S.); lyxnmyyzh@163.com (Z.Y.); 2 College of Environment and Resources, College of Carbon Neutrality, Zhejiang A&F University, Hangzhou 311300, China; 3 Zhejiang Provincial Key Laboratory of Carbon Cycling in Forest Ecosystems and Carbon Sequestration, Zhejiang A&F University, Hangzhou 311300, China; 4 Yongkang Forestry Technology and Seedling Extension Station, Yongkang 321300, China

**Keywords:** forest–medicinal plant agroforestry systems, plant functional traits, soil quality, rhizosphere processes, understory environment, ecosystem functions

## Abstract

Long-term monoculture management of plantation forests has caused declining soil fertility and biodiversity loss, a key bottleneck for sustainable forestry. Understory cultivation of medicinal plants, forming a multi-layered vertical structure, offers a promising pathway to improve land-use efficiency while balancing ecological protection and economic benefit. Using the framework “plant functional traits–soil processes–ecosystem functions,” this paper examines how forest–medicinal plant agroforestry systems affect soil quality and the understory environment. Aboveground traits (specific leaf area, leaf dry matter content) regulate soil carbon stability via litter input, while belowground traits (root architecture, exudates) drive soil structure and nutrient cycling through rhizosphere effects. These systems optimize soil pore structure through root penetration, influence organic matter accumulation via litter decomposition and root carbon input, and enhance nutrient availability via mycorrhizal symbiosis and organic acid secretion. However, poor long-term management may cause acidification, nutrient imbalance, and replant problems. Diversified carbon inputs also reshape microbial community structure, functional genes, and enzyme activities, while enhanced plant diversity regulates understory microclimate (light, temperature, moisture). Finally, the paper outlines future research directions and proposes a core “context-dependency” hypothesis, emphasizing that effect direction and intensity are nonlinearly modulated by forest type, medicinal species, and management duration.

## 1. Introduction

Forest ecosystems play a critical role in global carbon (C) cycling, with global forest C stocks estimated at approximately 870 ± 61 Gt C [1]. Sustainable forest management is therefore essential for regulating global C dynamics and maintaining ecological security [2]. In recent decades, forest resources in China have undergone continuous recovery, with substantial increases in both forest area and growing stock. Among these resources, plantation forests represent a considerable proportion of the national forest area, exceeding 80 million hectares [3]. However, long-term monoculture plantation management has increasingly resulted in problems such as soil fertility decline and biodiversity loss, making it difficult for conventional forest management practices to simultaneously achieve ecological conservation and economic benefits [4]. Under this context, understory-based forest management provides a promising approach to improving forest resource utilization efficiency. Among various practices, forest–medicinal plant agroforestry represents an important management model, which introduces medicinal plants into forest understories to establish multilayered vertical resource utilization structures [5]. Compared with monoculture plantations, understory agroforestry systems enable more efficient utilization of light, water, and nutrient resources through multidimensional resource partitioning [6]. Such systems not only enhance overall forest productivity and resource-use efficiency but also contribute to improving understory habitat conditions and strengthening ecosystem services [7]. Therefore, systematically evaluating the effects of forest–medicinal plant agroforestry systems on soil quality and the understory environment is of great scientific importance for improving forest resource utilization efficiency and maintaining soil health [8].

Forest–medicinal plant agroforestry systems (FMPAFS) refer to multilayered agroforestry systems established by introducing medicinal plants beneath tree stands [9,10]. Here, “forest” refers primarily to planted forests and woodlands rather than natural forests. In China, the practice of cultivating medicinal plants under forest cover has a long history, in which medicinal plants are intentionally introduced beneath trees for medicinal production and economic utilization. It should be noted that, from a broad ethnobotanical perspective, many plant species have been documented or traditionally reported to have medicinal uses [11]. To avoid ambiguity, the term “medicinal plant” in this review refers specifically to plant species that are intentionally cultivated and managed for medicinal production and economic purposes, including species recognized in official pharmacopoeias, such as the Chinese Pharmacopoeia [12]. This definition distinguishes the cultivated medicinal plants considered in FMPAFS from understory plants that may possess incidental or traditionally reported medicinal or non-timber forest product (NTFP) value but are not intentionally cultivated for medicinal production. FMPAFS can be established in plantations, economic forests, bamboo forests, and other suitable forest environments, depending on the ecological requirements of the cultivated medicinal plants [11]. Management practices may include site selection based on forest and soil conditions, seedling establishment or direct sowing, understory cultivation and management, and the harvesting of medicinal organs [11,13]. The intensity of management can vary among systems, ranging from relatively low-input cultivation to more intensive planting and management. Accordingly, FMPAFS may occur at different spatial scales, from smallholder or experimental plots to larger commercial production bases [13].

FMPAFS integrate ecological processes dominated by trees—including photosynthetic C fixation and litter inputs [1]—with processes driven by cultivated medicinal plants, including root activity, root exudation, and interactions with soil microbial communities [14]. Changes in understory vegetation and forest structure can also alter microclimatic conditions, including light availability and thermal conditions [15,16], and may influence understory vegetation composition and biodiversity [17]. However, continuous cultivation and intensive management may alter soil structure, nutrient availability, and microbial community composition [14,18,19]. These effects are particularly relevant for medicinal plants, as many cultivated species are harvested for belowground organs, including roots, rhizomes, and bulbs [20]. Consequently, their root functional traits and rhizosphere chemical inputs may influence soil structure, C and N cycling, and microbial community assembly [21,22,23]. Nevertheless, the mechanisms through which cultivated medicinal plants regulate soil processes via their functional traits, and how these changes propagate to the understory environment and ecosystem functions, remain insufficiently understood. A structured literature search was conducted using Web of Science, Google Scholar, and the China National Knowledge Infrastructure (CNKI), covering studies published from 2011 to 2026. The search used combinations of terms related to FMPAFS, understory medicinal plants, plant functional traits, soil processes, nutrient cycling, soil organic C, and microbial communities, as well as ecosystem functions. Relevant studies were identified and screened according to their relevance to the scope and objectives of this review, with particular emphasis on the links among medicinal plants, forest environments, plant functional traits, soil processes, and ecosystem functions.

Accordingly, this review focuses on the framework of “plant functional traits–soil processes–ecosystem functions” in FMPAFS. We synthesize the available evidence on how medicinal plant root characteristics and rhizosphere chemical inputs regulate soil physical structure, nutrient cycling, and microbial communities, from rhizosphere processes to ecosystem-scale effects. Furthermore, we aim to elucidate the mechanisms underlying the regulation of ecosystem functions and provide theoretical support for evaluating soil ecological impacts and optimizing sustainable forest–medicinal plant agroforestry management.

## 2. Plant Functional Traits of Understory Medicinal Plants

### 2.1. Key Plant Functional Traits and Their Ecological Functions

Plant functional traits are measurable morphological and physiological characteristics that reflect plant resource-acquisition strategies [24,25]. They can also be linked to soil ecological processes [26,27,28]. These traits are broadly divided into aboveground and belowground dimensions [21]. In FMPAFS, medicinal plants may show distinctive trait combinations related to their growth strategies and harvested organs. Root systems vary in architecture, spatial distribution, and turnover, reflecting distinct resource-use strategies [29]. Medicinal plants also have diverse specialized metabolites. Some of these compounds can enter the rhizosphere through root exudation and other pathways. They may influence rhizosphere microorganisms and plant–soil interactions [30]. These chemical traits therefore provide an additional dimension for understanding medicinal plant effects on soil. However, most current knowledge of trait–soil relationships comes from broader plant ecological, agricultural, and forest studies [31,32]. Direct evidence from FMPAFS remains limited. In particular, the links between medicinal plant functional traits and soil processes have not yet been sufficiently investigated.

#### 2.1.1. Aboveground Traits

Aboveground traits—including structural traits such as plant height and growth form, and leaf traits such as specific leaf area (SLA), leaf dry matter content (LDMC), and leaf nitrogen (N) content—jointly shape plant light-acquisition strategies and the quantity and chemical quality of litter returned to the soil [33,34,35]. In FMPAFS, medicinal plants vary considerably along these axes. Erect-form species (e.g., *Bupleurum chinense*) invest more in vertical stem growth, whereas prostrate, stoloniferous species (e.g., *Ophiopogon japonicus*) spread laterally and tolerate greater shading. These contrasting growth forms can therefore lead to differences in resource acquisition, vegetation structure, and understory microclimate.

SLA and LDMC likewise vary widely among cultivated medicinal species, and this variation can shape soil C dynamics through different litter-mediated pathways rather than a single “faster decomposition, less C” relationship. Species with high SLA often produce relatively labile litter that is more readily processed by soil microorganisms. Microbial processing of plant-derived C can promote microbial necromass formation and its subsequent stabilization as mineral-associated organic matter (MAOM), which represents a relatively persistent SOC pool [36]. In contrast, low-SLA, lignin-rich species tend to produce more slowly decomposing litter that can contribute to particulate organic matter (POM) formation. Compared with MAOM, POM is less protected by mineral association and can be more vulnerable to disturbance-induced mineralization. Thus, the relative contribution of medicinal plant litter to different SOC pools, rather than decomposition rate alone, can influence long-term SOC stability.

In FMPAFS, these aboveground traits are particularly relevant because medicinal plants grow beneath tree canopies, where light, temperature, and moisture conditions differ from those in open habitats. Variation in these traits can influence resource acquisition, litter inputs, and understory microclimate, thereby linking medicinal plant functional strategies to soil processes.

#### 2.1.2. Belowground Traits

In FMPAFS, understory medicinal plants can be broadly distinguished by contrasting root architectures. Taproot-type species, such as *Panax notoginseng* and *Panax ginseng*, develop a dominant primary root that serves as their principal storage and harvested organ [37]. Their root systems therefore emphasize vertical development and biomass allocation to the primary root. In contrast, fibrous-root-type species such as *Sarcandra glabra* lack a dominant primary root and instead develop a fibrous root system. Root morphological traits in this species—including root length, surface area, and volume—have been shown to respond substantially to light spectral quality under controlled conditions [38]. Because forest canopies selectively filter light spectra in addition to reducing overall irradiance, this sensitivity suggests that canopy-mediated changes in light quality may represent an underexplored driver of root morphology in understory-grown medicinal plants, though this has not yet been directly tested in FMPAFS.

Root architecture is also plastic in response to resource availability. For example, under elevated N supply, *P. notoginseng* reduced fibrous root number, fibrous root length, and total root length while increasing the relative allocation of biomass aboveground [39]. This response indicates that belowground foraging investment can vary with nutrient availability rather than remaining fixed. Root nutrient concentrations, particularly root N and phosphorus, can further indicate plant nutrient acquisition strategies and resource-use trade-offs [40]. Beyond architecture, morphology, and nutrient concentration, mycorrhizal association represents another important belowground trait dimension in FMPAFS. Arbuscular mycorrhizal fungi (AMF) can extend the effective absorptive area of plant roots and contribute to nutrient acquisition.

It should be noted that, unlike the increasingly detailed root trait datasets available for forest and grassland species [40], quantitative measurements of SRL, RTD, and root diameter for medicinal plants remain scarce. Most available studies report root characteristics in agronomic or morphological terms rather than within a formal trait-economics framework. Applying this framework more systematically to FMPAFS root systems therefore represents an important direction for future research.

### 2.2. Core Traits Associated with Root-Mediated Chemical Modification: Composition and Secretion Patterns of Root Exudates

Root exudates generally refer to organic compounds actively or passively released by plant roots into the surrounding environment during root growth. These compounds include low-molecular-weight substances, such as organic acids, amino acids, soluble sugars, soluble proteins, peptides, and plant hormones; high-molecular-weight compounds, including root cap cells, mucilage, mucus substances, and extracellular enzymes; as well as cellular debris, such as root hair fragments and cell residues [41]. Through continuous chemical release, plant roots establish complex chemical gradients within the rhizosphere soil [30]. This chemical output represents a key component of root functional traits and directly influences soil microbial assembly, nutrient mineralization, and ecosystem functions [42]. In FMPAFS, the distinct metabolic profiles of medicinal plants can introduce additional sources of root-derived compounds into plantation soils, potentially modifying rhizosphere conditions relative to timber monocultures.

#### 2.2.1. Primary Metabolites: Sugars, Organic Acids, and Amino Acids

Primary metabolites provide selective C and nutrient sources that influence rhizosphere microbial community structure [43,44,45]. Sugars provide readily available C substrates for microbial growth and can stimulate soil organic matter (SOM) decomposition [46], whereas organic acids can mobilize mineral-bound nutrients through chelation and modify rhizosphere pH [47]. Amino acids can serve as both C and N sources and may influence microbial community composition and nutrient transformations [48]. Because these metabolites are rapidly assimilated or transformed in the rhizosphere [49], their in situ concentrations remain difficult to quantify, and direct quantitative comparisons among medicinal plant species remain limited.

#### 2.2.2. Secondary Metabolites: Saponins, Flavonoids, Alkaloids, and Volatile Oils

Secondary metabolites represent a distinctive chemical dimension of many medicinal plants and can influence rhizosphere microbial interactions [50,51]. For example, *Panax* species produce saponins such as ginsenosides and notoginsenosides, some of which can be released from roots and detected in rhizosphere soils [52]. Species such as *Coptis chinensis* and *Berberis* produce berberine and related alkaloids with antimicrobial activity [53]. These compounds may modify microbial community composition in medicinal plant rhizospheres. Under continuous cultivation, some root-derived secondary metabolites may accumulate in the rhizosphere and contribute to autotoxicity and changes in rhizosphere microbial community structure [14]. Thus, secondary metabolites may represent a chemical pathway linking root traits with rhizosphere microbial processes in FMPAFS. However, current evidence does not establish that medicinal plants generally release greater amounts of secondary metabolites than other understory plants; their occurrence and ecological effects depend on plant species, metabolite composition, cultivation duration, and soil conditions.

#### 2.2.3. Rhizosphere Enzyme Activities and Redox Capacity

Root-derived C inputs from medicinal plants can influence microbial activity and associated enzyme production, potentially altering C, N, and P transformation processes in the rhizosphere [54,55]. In FMPAFS, these effects provide a functional link between medicinal plant root activity and soil nutrient cycling beneath plantation canopies. Root respiration and exudate inputs may also modify local redox conditions through processes such as radial oxygen loss, whereby oxygen diffusing from root tissue into the rhizosphere drives the oxidation of reduced, soluble forms of Fe and Mn into less soluble oxidized forms that can precipitate as plaques on root surfaces [56]. This mechanism has been most extensively documented in wetland and paddy systems; its relevance to FMPAFS, where soil moisture conditions differ substantially, has not yet been directly examined.

## 3. Mechanisms Underlying the Regulation of Soil Properties by FMPAFS

### 3.1. Effects on Soil Physical Properties

Soil bulk density and pore structure in FMPAFS are jointly regulated by the mechanical and biochemical effects of root traits in different types of medicinal plants [57]. Taproot-type plants penetrate compact soil layers and create stable macropore channels, thereby facilitating deep soil aeration and water infiltration. This mechanism of root-induced macropore formation is well established in agriculture and grassland systems [31,58]; direct evidence within FMPAFS is limited to a small number of studies on specific taproot species [59,60]. For example, after two years of understory cultivation of *Panax notoginseng* in a plantation, bulk density in deep soil layers (>30 cm) was significantly lower than that under conventional farmland cultivation, accompanied by an increase in preferential flow pathways [59,60]. In contrast, fibrous-root-type plants primarily influence surface soils by enhancing microaggregate stability, reducing bulk density in the topsoil, and accelerating soil structural renewal [61,62]. Gan et al. reported that cultivation of *Sarcandra glabra* in a *Cunninghamia lanceolata* plantation significantly reduced topsoil bulk density while increasing total porosity and capillary porosity, with these regulatory effects being mainly confined to shallow soil layers [63]. Collectively, medicinal plants optimize soil pore structure in FMPAFS, and the resulting changes further regulate soil water infiltration, retention, and redistribution processes [64].

Soil aggregates are the fundamental structural units of soil, and their size distribution and stability determine the persistence of pore structure and soil resistance to erosion [65]. In FMPAFS, root systems, litter inputs, and rhizosphere microbial activities collectively contribute to soil aggregate formation. Organic binding agents, including root-derived polysaccharides, mucilaginous substances, and glomalin-related soil proteins (GRSP) produced by arbuscular mycorrhizal fungi, enhance adhesion among soil particles and promote formation of macroaggregates [66]. Meanwhile, the physical entanglement of roots and fungal hyphae further improves aggregate stability. Zeng et al. reported that, after three years of a fruit tree–*Hypericum monogynum*—AFS in a karst region, the proportion of macroaggregates (>2 mm) increased significantly, whereas the soil fractal dimension decreased, indicating enhanced soil structural stability and reduced soil erodibility [67]. This example illustrates how the introduction of medicinal plants with diverse root traits can increase the input of organic binding agents and root–hyphal entanglement, thereby improving aggregate stability relative to monoculture plantations.

However, under continuous cropping conditions, the accumulation of autotoxic compounds suppresses the activity of beneficial microorganisms and reduces the production of binding substances, such as root-derived polysaccharides and extracellular polymeric substances (EPS). Consequently, macroaggregates are disrupted and the proportion of microaggregates increases, indirectly reducing aggregate stability and accelerating soil compaction and structural degradation [18,61,68]. Taken together, the taproot- and fibrous-root-mediated pore formation described above, alongside the risk of aggregate breakdown under continuous cropping, indicate that the net effect of medicinal plant introduction on soil physical structure in FMPAFS depends on the balance between root-mediated soil-building processes and continuous cropping. Therefore, the evolution of soil aggregates in FMPAFS is fundamentally regulated by the combined effects of plant roots, biological binding processes, and allelochemicals. Changes in aggregate stability can thus serve as an important indicator for evaluating soil structural health in these systems.

### 3.2. Effects on Soil Chemical Properties

#### 3.2.1. SOM Accumulation and Carbon Cycling

The cultivation of understory medicinal plants influences SOC dynamics primarily through two pathways—litter inputs and root-derived carbon inputs—both of which are jointly regulated by leaf and root traits [34,69]. Rather than a single “faster decomposition, less carbon” relationship, these inputs shape SOC accumulation through two distinct stabilization pathways, consistent with the microbial efficiency–matrix stabilization (MEMS) framework [36].

Leaf traits determine litter quality and, consequently, the pathway through which litter carbon is processed. Medicinal plants with high specific leaf area (SLA), such as peppermint (*Mentha* × *piperita*) and *Houttuynia cordata*, typically produce labile, nutrient-rich litter. Because this substrate is readily assimilated by soil microorganisms, its carbon can be efficiently converted into microbial biomass and necromass, which is subsequently stabilized as MAOM—often the most persistent SOC fraction—through interactions with soil minerals [36]. In contrast, medicinal plants with low SLA and higher lignin content produce more slowly decomposing litter that accumulates primarily as POM; while POM can persist visually and chemically for extended periods, it lacks mineral protection and remains more vulnerable to disturbance-induced mineralization than MAOM [36]. Consequently, the relative contribution of medicinal plant litter to these two pools—rather than decomposition rate alone—determines its net effect on long-term SOC stability.

Root-derived carbon follows a related but distinct logic. Living roots continuously release labile, readily decomposable compounds through rhizodeposition, which can, through microbial processing, contribute disproportionately to microbial necromass formation and subsequent MAOM stabilization [69]. This is consistent with direct evidence that root-litter decomposition, compared with leaf-litter decomposition, more strongly promotes the accumulation of mineral-associated organic carbon and the MAOC/POC ratio, indicating a greater relative contribution of belowground inputs to stable soil carbon pools [70]. Soluble compounds released by roots also stimulate microbial activity and promote the formation of stable soil aggregates, particularly through AMF, enhancing the physical protection of SOC within aggregates [71,72].

In FMPAFS, the introduction of medicinal plants has been associated with increases in SOC content. For example, understory cultivation of *Sarcandra glabra* and *Polygonatum cyrtonema* in *Cunninghamia lanceolata* plantations has been shown to significantly increase SOC content [63]. However, these effects are not invariably positive. Under continuous cropping, large quantities of labile carbon released through root exudation may accelerate the mineralization of pre-existing SOM—a priming effect that can result in a net loss of SOC under certain conditions [73,74]. In addition, soil disturbance and fertilization practices associated with some FMPAFS management—such as tillage during planting and harvesting—may disrupt soil physical structure and accelerate organic matter mineralization; such effects are well documented in conventional agricultural systems, including no-till versus tillage comparisons [75] and long-term paddy fertilization studies [76], but the extent to which these mechanisms operate similarly under the generally lower-intensity management typical of FMPAFS remains poorly characterized. Therefore, the effects of medicinal plant cultivation on SOM exhibit a dual nature and depend on multiple factors, including medicinal plant species, cultivation duration, and management intensity [77,78,79].

#### 3.2.2. Soil Nutrient Availability

Understory medicinal plants influence soil nutrient cycling through multiple pathways, including mycorrhizal associations and the chemical mobilization of nutrients mediated by low-molecular-weight compounds released from roots [80,81]. These processes contribute to the development of spatially differentiated nutrient acquisition patterns within FMPAFS. Mycorrhizal associations represent one of the primary mechanisms by which plants acquire nutrients with low mobility in soils [81]. In FMPAFS, some medicinal plants establish symbiotic relationships with arbuscular mycorrhizal fungi (AMF), thereby expanding the effective absorptive zone of the root system and enhancing the acquisition of phosphorus (P) and other poorly mobile nutrients. For example, in a greenhouse experiment, inoculation of *Polygonum cuspidatum* with AMF increased plant height and root biomass by enhancing P uptake through fungal hyphae [82], providing indirect evidence for the contribution of the mycorrhizal pathway to P acquisition. Similarly, in *Panax ginseng*, AMF colonization improved biomass production and saponin accumulation under varying soil P availability, with *Funneliformis mosseae* particularly enhancing P absorption efficiency at low-P levels [83]. During hyphal growth, AMF can also recruit and enrich hyphosphere bacteria with phosphate-solubilizing capabilities, thereby promoting the mineralization and mobilization of soil organic P [84]. Together, these interactions constitute a nutrient cycling pathway characterized by mycorrhizal-mediated nutrient acquisition.

Root-secreted organic acids represent a second nutrient-mobilizing pathway, well documented in agricultural crops such as maize [32]. Evidence from *Panax ginseng* rhizospheres is also emerging: root-released organic acids and associated phosphate-solubilizing microbes, including *Burkholderia vietnamiensis*, contribute to P mobilization by secreting malic and tartaric acid, which chelate Ca^2+^, Fe^3+^, and Al^3+^ [85], though this evidence derives from ginseng cultivation generally rather than forest-understory conditions specifically. The fate of chelated metal ions varies with organic acid type and concentration: in dryland soils, citrate and malate increase short-term Ca and Mg solubility, whereas oxalate can precipitate as calcium oxalate at higher concentrations, reducing Ca availability; Fe and Mn chelation, by contrast, typically maintains these micronutrients in plant-available forms [86]—thresholds that have not yet been tested in FMPAFS soils.

These acquisition strategies may generate spatial differentiation of nutrient uptake throughout the soil profile [87]. Differences in root distribution among species often produce vertical stratification of nutrient use, reducing interspecific competition and improving system-level nutrient-use efficiency [88,89], although resulting spatial heterogeneity may also cause localized imbalances affecting plant growth [87]. Overstory tree species composition further shapes soil nutrient dynamics in FMPAFS. Intercropping *Bletilla striata* with fruit trees (apple, pear, peach) increases SOM, total P, total potassium, and bacterial diversity, though the specific nutrients enhanced vary by tree species and may carry risks such as soil acidification [90]; similar tree-species-dependent differences in SOM and available P have been reported for Ophiopogon japonicus [91]. In *Pinus elliottii* plantations, the P. elliottii–*Alpinia hainanensis*–*Spatholobus suberectus* pattern enhanced acid phosphatase, neutral protease, and urease activities and supported the highest microbial richness among tested patterns, with SOC and total N as key drivers [92]. Teak (*Tectona grandis*) intercropped with *Alpinia katsumadai* and *Amomum longiligulare* increased SOM, total K, and available N [93], while *Idesia polycarpa* intercropped with *Salvia rosmarinus* showed the highest SOM and available potassium among five tested patterns [94]. Collectively, these findings indicate that tree species composition is an important determinant of soil nutrient dynamics in FMPAFS, though the magnitude and direction of effects depend on the specific overstory–understory combination, soil depth, and local edaphic conditions.

#### 3.2.3. Changes in Soil pH

Soil pH is a key factor governing soil nutrient availability and microbial activity [71]. Understory medicinal plants regulate soil pH by influencing the rhizosphere environment and soil processes through nutrient uptake, the release of low-molecular-weight compounds from roots, and litter decomposition, thereby further affecting nutrient availability and microbial activity [57,95]. From the perspective of nutrient uptake, the acquisition of different N forms exerts distinct effects on rhizosphere pH. Uptake of ammonium (NH_4_^+^) is generally accompanied by the release of H^+^ from plant roots, leading to rhizosphere acidification. For example, cultivation of *Polygonatum cyrtonema* under Chinese fir plantations significantly increased soil ammonium concentrations while reducing total P and available P contents [63]. In contrast, nitrate (NO_3_^−^) uptake may promote rhizosphere alkalization through the release of OH^−^ or HCO_3_^−^ [96]. Consequently, differences in N-use strategies among medicinal plant species partly determine the direction and magnitude of changes in soil pH [97,98]. In FMPAFS, studies of cultivated medicinal plants have reported that some species preferentially utilize ammonium N; therefore, long-term cultivation may be associated with a gradual decline in soil pH [14,99]. The release of low-molecular-weight compounds from roots represents another important mechanism regulating soil pH. Plant roots can secrete low-molecular-weight organic acids, such as citric acid and malic acid, which mobilize mineral-bound P through chelation of Ca^2+^, Fe^3+^, and Al^3+^ ions, as well as competition for adsorption sites. This mechanism has been well documented in crops such as maize [32]. Based on their well-developed root systems and strong exudation capacity, understory medicinal plants are expected to promote P mobilization through similar mechanisms; however, direct experimental evidence remains scarce. In addition, the release of secondary metabolites, particularly phenolic acids, may alter the chemical environment of the rhizosphere and thereby exert persistent effects on soil pH [100,101].

Litter decomposition also plays an important role in the long-term regulation of soil pH. During decomposition, litter releases organic acids and various inorganic ions, thereby altering soil pH [102]. In general, litter rich in N and soluble components decomposes rapidly, releasing organic acids and ammonium over a relatively short period and consequently causing a temporary decline in soil pH. In contrast, litter containing high proportions of structural compounds, such as lignin and cellulose, decomposes more slowly. During this process, the continuous release of humic substances enhances the soil buffering capacity, resulting in more persistent and moderate regulation of soil pH [103,104]. Furthermore, litter decomposition can influence the accumulation of SOM, thereby modifying soil buffering capacity and ultimately regulating the magnitude of soil pH fluctuations [105,106].

#### 3.2.4. Potential Ecological Risks

In FMPAFS, appropriate applications of fertilizers and pesticides help maintain the growth of medicinal plants, improve productivity, and, to some extent, enhance soil nutrient supply [107,108]. However, excessive inputs or inappropriate management practices may disrupt soil nutrient balance and consequently give rise to a range of ecological risks [109]. Long-term excessive N fertilization may suppress soil nitrogenase activity, thereby reducing biological N fixation [110], while excessive phosphorus application beyond plant uptake capacity can increase the risk of P leaching, not only decreasing on-site nutrient-use efficiency but also raising the potential for downstream eutrophication in adjacent water bodies [111]. In addition, excessive or improper pesticide application may not only result in pesticide residues in the soil but also alter the composition of soil microbial communities, thereby affecting soil ecological processes [112]. Under continuous cropping conditions, these problems are often exacerbated because of the combined effects of nutrient accumulation and the buildup of harmful substances.

Furthermore, some medicinal plants possess the capacity to absorb and accumulate heavy metals. Under conditions of poor soil environmental quality or increased external inputs of heavy metals, these elements may accumulate in plant tissues, thereby posing potential risks to the quality and safety of medicinal materials [113,114]. Therefore, comprehensive assessments of heavy metal migration and transformation from the perspective of the soil–plant system are essential for evaluating the ecological sustainability of FMPAFS. More broadly, these management-related effects indicate that management intensity is not merely a source of ecological risk but may also regulate the expression and magnitude of trait-mediated ecological effects in FMPAFS.

### 3.3. Effects on Soil Biological Properties

#### 3.3.1. Soil Microbial Communities

The composition and structure of soil microbial communities in FMPAFS are primarily influenced by plant species composition [115,116]. Sugars, organic acids, and amino acids released from plant roots provide readily available C sources for soil microorganisms, generating pronounced rhizosphere effects that promote the enrichment of specific microbial taxa [84,117]. Because different microorganisms differ in their capacities to utilize C substrates, the composition of low-molecular-weight compounds in root exudates directly shapes the composition of bacterial and fungal communities [116,118]. For example, studies conducted in understory cultivation systems of *Panax notoginseng* and *Panax ginseng* showed that the rhizosphere was enriched with bacterial taxa involved in nutrient cycling, including N-fixing and phosphate-solubilizing bacteria, thereby improving soil nutrient transformation efficiency [119,120]. In addition, secondary metabolites released by roots modify soil microbial communities through chemical regulation [121]. For example, phenolic acids, terpenoids, and alkaloids can selectively suppress potential soil pathogens by disrupting cell membrane integrity or inhibiting key enzymatic activities [122]. At the same time, these compounds may also function as signaling molecules or low-concentration substrates that facilitate the recognition and colonization of beneficial symbiotic microorganisms, particularly arbuscular mycorrhizal fungi (AMF) [123,124].

Litter inputs from understory medicinal plants also play an important role in shaping microbial community composition [125]. Compared with monoculture forest stands, FMPAFS possess a more diverse plant community, resulting in substantial variation in litter chemical composition [126]. Consequently, a greater diversity of organic substrates is introduced into the soil. These diverse C sources provide ecological niches for microorganisms with different functional traits, thereby enhancing microbial diversity and increasing the stability of microbial interaction networks [127].

Furthermore, FMPAFS regulate the functional composition of soil microbial communities by altering resource quality and decomposition strategies [128,129]. On the one hand, an increased supply of readily decomposable substrates generally favors fast-growing bacterial taxa characterized by r-strategist life-history strategies [130]. On the other hand, litter rich in lignin and other structurally complex organic compounds promotes the proliferation of fungi and cellulose-degrading microorganisms, thereby shifting the relative dominance of bacterial and fungal communities [131,132]. In addition, several studies have reported that understory medicinal plant cultivation increases the relative abundance of microbial functional groups associated with organic matter decomposition and nutrient transformation, including cellulose-degrading microorganisms and microorganisms involved in N transformation, thereby accelerating litter decomposition and nutrient mineralization [133,134].

Overall, by increasing the diversity of plant functional traits and enhancing the diversity and heterogeneity of soil C inputs, FMPAFS drive the restructuring of soil microbial communities in both taxonomic composition and functional potential, ultimately influencing nutrient cycling and other ecosystem processes [135,136].

#### 3.3.2. Soil Enzyme Activity

Soil enzyme activity is an important indicator of soil nutrient transformation and biological activity [137,138]. As the catalytic mechanisms and ecological functions of major soil enzymes have been discussed in the previous section, they are not described in detail here. In FMPAFS, soil enzyme activity varies with medicinal plant species and cultivation practices, resulting in distinct patterns among different systems [128,139]. For example, in understory cultivation systems of *Panax notoginseng*, acid phosphatase activity has been identified as a particularly sensitive indicator [140]. In Chinese fir plantations intercropped with *Sarcandra glabra*, the activities of sucrase and urease increased and were positively correlated with soil total N and ammonium N contents [141]. Similarly, in *Camellia oleifera* plantations intercropped with five medicinal plant species, catalase and acid phosphatase activities were enhanced in plots cultivated with *Clinacanthus nutans* and *Curcuma longa* [128]. Overall, the enzyme activities that respond differ among FMPAFS, largely owing to differences in litter inputs, the release of low-molecular-weight compounds in root exudates, and nutrient acquisition strategies among plant species [142,143]. In these systems, medicinal plants increase organic matter inputs and root-derived C inputs, thereby providing additional substrates for soil microorganisms, which stimulates microbial growth and metabolism to promote the production of extracellular enzymes involved in nutrient cycling [142,144]. Furthermore, differences in plant demand for nutrients such as N and P may influence microbial resource allocation strategies and the expression of enzyme activities [137,145,146,147]. Therefore, variations in soil enzyme activity among different FMPAFS reflect the integrated effects of plant functional traits, microbial activity, and soil environmental conditions, highlighting the coordinated regulation of soil nutrient cycling by plant species composition in these agroforestry systems.

## 4. Effects of FMPAFS on the Understory Environment

FMPAFS not only modify the structure of understory vegetation but also influence the understory microclimate, biological community composition, and forest ecosystem functions through changes in plant functional traits [148,149]. The introduction of medicinal plants effectively adds an additional functional layer between the overstory trees and the soil within the existing forest ecosystem. The aboveground functional traits of medicinal plants alter light partitioning and microclimatic conditions, whereas their root architecture and root exudates further regulate soil microbial activity and nutrient cycling [150]. Therefore, the environmental effects of FMPAFS are fundamentally ecological processes driven by plant functional traits.

### 4.1. Effects on Understory Plant Diversity

In FMPAFS, the introduction of medicinal plants often modifies the structure of understory vegetation and significantly influences the species richness and composition of plant communities [151,152]. Compared with monoculture plantations, these systems develop a multilayered vegetation structure that increases niche dimensionality, allowing plant species with different life forms to occupy distinct ecological niches under varying light and resource conditions. This structural complexity can alleviate direct interspecific competition to some extent [153,154].

Aboveground functional traits play a critical role in this process. As discussed in Section 2, functional traits such as leaf traits and plant height influence canopy structure and the distribution of light resources, thereby affecting the establishment and growth of different plant species [149,155]. Moderate shading and greater light heterogeneity favor the persistence of shade-tolerant species and promote the coexistence of species with contrasting ecological strategies within plant communities [156,157]. Consequently, increased vegetation structural complexity in understory cultivation systems is often accompanied by higher species richness and community evenness.

In addition to aboveground processes, belowground interactions also contribute to community assembly. Through nutrient uptake and the release of root exudates, plant roots modify the rhizosphere environment, thereby influencing soil microbial communities and nutrient cycling [57]. These changes generate spatial heterogeneity in soil resources at relatively small scales, which in turn alters competitive relationships among plant species and contributes to the regulation of community composition [87,158,159].

Furthermore, forest–medicinal plant management may indirectly affect understory animal communities by providing more structurally complex habitats [160]. Multilayered vegetation creates a wider range of habitat niches for insects, birds, and other small animals while simultaneously increasing food resource availability, thereby contributing to the maintenance of relatively high levels of biodiversity [161,162,163]. From an ecosystem perspective, therefore, FMPAFS not only reshape plant community structure but may also influence organisms at higher trophic levels through multiple ecological processes [162].

### 4.2. Regulation of Understory Microclimate

FMPAFS regulate the understory microclimate by modifying vegetation vertical structure, thereby affecting radiation transfer, energy allocation, and water cycling processes within forest ecosystems [59,164]. In recent years, forest microclimate has become an important research focus in forest ecology and global change studies. Previous studies on forest microclimates have generally demonstrated that forest canopies create more stable thermal and atmospheric moisture conditions in understory environments compared with open areas through processes such as shading, transpiration, and litter layer coverage. Transpiration releases water vapor into the understory atmosphere, while canopy shading and litter coverage reduce heat and moisture loss, thereby contributing to a relatively humid and stable understory microclimate. This phenomenon is known as the microclimate buffering effect, in which forest vegetation reduces understory temperature during hot periods and slows heat loss during cold periods, resulting in substantially lower temporal fluctuations in temperature and water availability compared with exposed environments [165]. By introducing an additional medicinal plant layer beneath the overstory canopy, FMPAFS further increase vegetation structural complexity and may enhance the buffering capacity of understory microclimates, thereby creating more suitable environmental conditions for medicinal plant growth and contributes to the stable functioning of forest ecosystems.

#### 4.2.1. Effects on Understory Water Availability

The multilayer vegetation structure formed by FMPAFS provides an important foundation for regulating understory water conditions. Vegetation intercepts precipitation and reduces solar radiation input, while canopy structure and plant transpiration modify near-ground water and energy fluxes [166]. The forest canopy can reduce near-surface wind speed and constrain atmospheric mixing [167], while the litter layer can retain water and reduce forest-floor evaporation [168,169]. These processes contribute to the maintenance of a relatively stable and humid understory environment. However, the relationship between forest canopy closure and understory moisture conditions is not simply a linear pattern in which higher canopy closure results in greater humidity. For example, Lenk et al. found that increasing canopy openness in a European mixed floodplain forest increased soil and air temperature fluctuations, while soil moisture was also moderately higher in more open areas, which the authors attributed partly to reduced tree transpiration and precipitation interception [170]. Canopy cover can also influence the spatial distribution and availability of understory soil moisture by modifying microclimatic conditions and water fluxes [171,172]. In addition, the stratified distribution and resource acquisition of different medicinal plant root systems contribute to optimizing water use patterns, reducing interspecific water competition, and improving overall water use efficiency and drought resilience within the system [173,174,175].

#### 4.2.2. Regulation of Understory Temperature

FMPAFS regulate understory temperature by modifying canopy structure and vegetation composition [172,176]. Forest canopies reduce incoming shortwave solar radiation and dissipate heat through plant transpiration, thereby lowering understory air temperature, reducing diurnal temperature fluctuations, and creating a more stable thermal environment [165]. Such a stable thermal environment can alleviate the stress of extreme high temperatures on medicinal plant growth and provide relatively stable conditions for plant physiological processes and ecosystem functions in the understory. Meanwhile, forest thermal environments are closely associated with plant composition and plant functional traits. Based on terrestrial laser scanning data, Wang et al. found that tree species composition had a stronger influence on temperature buffering capacity than some canopy structural parameters in young plantations, suggesting that plant functional traits may directly regulate understory temperature more effectively than structural characteristics alone [177]. This finding is consistent with the mechanisms discussed in Section 2, where leaf traits and plant height influence light acquisition and transpiration capacity. It further indicates that the functional traits of medicinal plants themselves may directly contribute to the regulation of understory thermal conditions, rather than acting solely through modifications of canopy structure. The stable thermal environment established by FMPAFS further influences surface energy exchange processes. Multilayer vegetation collectively reduces solar radiation input and decreases heat accumulation at the soil surface [178,179]. Meanwhile, litter coverage buffers heat exchange between the soil surface and the external environment, thereby reducing soil temperature fluctuations and providing relatively stable conditions for root growth, soil biological activity, and nutrient cycling [180,181,182,183]. Furthermore, forest temperature buffering capacity is closely linked with water availability, showing clear nonlinear coupling between thermal and hydrological conditions. Greiser et al. found that temperature buffering capacity increased with improving soil moisture conditions; however, this response became more sensitive under drought conditions, whereas under sufficient water availability, the buffering effect gradually approached saturation and was jointly regulated by canopy structure and site conditions [180]. These findings indicate that the stability of understory thermal environments depends not only on canopy structure but also on the water status of forest ecosystems. This conclusion is consistent with the humidity regulation effects discussed above, suggesting that temperature and moisture regulation in FMPAFS are interconnected and jointly driven processes.

## 5. Conclusions and Perspectives

### 5.1. Conclusions

In this review, we systematically synthesized existing research on the ecological effects of FMPAFS (Figure 1), focusing on their effects on understory vegetation, soil properties, microbial communities, C and nutrient cycling, and understory microclimate. The available evidence indicates that FMPAFS can modify multiple ecological processes through changes in vegetation structure, root-derived inputs, soil environments, and microbial communities. However, these ecological effects often exhibit inconsistent or even contrasting patterns across different systems and management contexts. These differences do not necessarily indicate contradictions among research findings but rather suggest that the direction and magnitude of ecological effects are dependent on contextual conditions.

As an important model of forest multifunctional management, FMPAFS has profound strategic significance for improving forest resource utilization efficiency, ensuring the supply of medicinal plant resources, and promoting rural revitalization. Meanwhile, these systems influence C and nutrient cycling, as well as related soil and ecosystem functions, by altering understory vegetation structure, soil environments, and microbial community composition. Therefore, under the framework of ecological conservation and sustainable utilization, systematically evaluating the ecological effects of FMPAFS and exploring management strategies that optimize both ecological and economic benefits have become important research directions in forest ecology.

Existing studies have shown that the ecological effects of FMPAFS often exhibit inconsistent or even contradictory patterns. For example, increases in soil organic C accumulation may coexist with C mineralization losses, while enhanced microbial diversity may occur alongside risks of continuous cropping obstacles. These differences do not necessarily indicate contradictions among research findings but rather suggest that the direction and magnitude of ecological effects are dependent on management contexts. Specifically, the same ecological process may exhibit threshold responses or nonlinear shifts depending on factors such as forest type, medicinal plant species, management duration, management intensity, climatic conditions, and initial soil properties. For instance, short-term forest–medicinal plant intercropping may enhance soil microbial diversity and nutrient availability through root exudate inputs and litter deposition. However, with prolonged cultivation duration, intensified resource competition and convergence of root exudate characteristics may reduce functional redundancy within microbial communities, thereby increasing the risk of continuous cropping obstacles. Moreover, management intensity can modify the expression and magnitude of these trait-mediated effects. Practices such as site preparation, fertilization, pesticide application, weed control, and harvesting may alter soil disturbance, resource availability, and plant-derived C and nutrient inputs. In particular, root harvesting can directly remove belowground biomass and disturb soil structure, potentially weakening or overriding beneficial effects associated with medicinal plant functional traits. Based on this perspective, this review proposes the scientific hypothesis that the ecological effects of FMPAFS exhibit context dependency, suggesting that their response patterns vary nonlinearly or across thresholds along gradients of key contextual variables rather than remaining constant.

Overall, FMPAFS represents not only an important pathway for promoting multifunctional utilization of forest resources but also an effective approach for achieving coordinated development of ecological conservation and forestry economies. Future research should further optimize forest–medicinal plant management practices under ecological theoretical frameworks. Accordingly, management intensity should be considered as an integral driver within the proposed framework, together with forest conditions and medicinal plant functional traits, because management practices may regulate the strength and direction of trait-mediated effects on soil processes and ecosystem functions. By strengthening long-term ecological monitoring and implementing science-based management strategies, it will be possible to achieve synergistic improvements in economic, ecological, and social benefits, thereby promoting the sustainable development of understory medicinal plant industries.

### 5.2. Perspectives

Although substantial progress has been made in understanding the ecological effects of FMPAFS, several limitations remain, and future research should be further advanced in the following aspects.

First, long-term monitoring studies should be strengthened. Most current studies are based on short-term experiments or single-time surveys, which limits our ability to reveal the temporal dynamics of ecological effects in FMPAFS. However, forest ecosystems inherently exhibit strong temporal dynamics, and understory vegetation structure, soil nutrient cycling, and microbial community composition often require extended periods to develop stable patterns. The lack of long-term monitoring data currently restricts comprehensive understanding of the persistent effects and long-term trajectories of FMPAFS on ecological processes. Therefore, long-term ecological monitoring should be promoted through the establishment of permanent observation plots to clarify the dynamic changes in ecological effects and provide reliable data foundations for ecological model development.

Second, the mechanisms underlying context dependency should be further explored and validated across regions. Current studies mainly focus on changes in individual components, such as soil nutrient contents, enzyme activities, and microbial community structures, while the integrated relationships among plant roots, soil microorganisms, and soil metabolic processes remain poorly understood. This limitation makes it difficult to identify the mechanisms driving context-dependent responses. In recent years, advances in multi-omics approaches, including metagenomic sequencing and untargeted metabolomics, have provided new opportunities to address this issue. Some studies have attempted to integrate microbial community composition, functional genes, and soil metabolite profiles to reveal mechanisms underlying soil microecological reconstruction and C and nutrient cycling in understory cultivation systems. However, such studies remain limited, particularly those focusing on the coordinated interactions among plants, soil, microorganisms, and metabolites in FMPAFS.

Meanwhile, most existing studies are still concentrated on individual medicinal plant species or localized cultivation practices, with insufficient comparative research across regions and ecosystem types. This limitation reduces the general applicability of current findings and prevents comprehensive validation of the proposed context dependency hypothesis. Future research should systematically establish functional trait spectra of understory medicinal plants, develop quantitative models linking plant functional traits, soil microorganisms, and ecosystem functions, and integrate multi-omics approaches, including metagenomics, metabolomics, and stable isotope techniques. These efforts should facilitate the development of a conceptual framework centered on the interactions among environmental conditions–management practices–plant functional traits–soil microecosystems–ecological processes–ecological effects, thereby enabling systematic evaluation of the context dependency hypothesis and providing theoretical support for precision configuration and adaptive management of FMPAFS across different regions.

Furthermore, the integrated evaluation framework for ecological and economic benefits of FMPAFS requires further improvement. Current studies mainly evaluate individual indicators, such as medicinal plant yield or soil fertility changes, while systematic quantification of trade-offs among ecosystem services, economic benefits, and ecological risks remains insufficient. Therefore, multi-objective optimization models should be developed to achieve coordinated improvements in ecological and economic outcomes. Future studies could combine ecosystem service assessment approaches with ecological economic models to conduct scenario simulations and optimization analyses of different forest–medicinal plant configurations, thereby providing scientific support for sustainable management. Meanwhile, under the framework of ecological restoration and sustainable utilization, future research should explore Nature-based Solutions by constructing multilayered and multispecies FMPAFS that mimic near-natural forest structures.

## Figures and Tables

**Figure 1:**
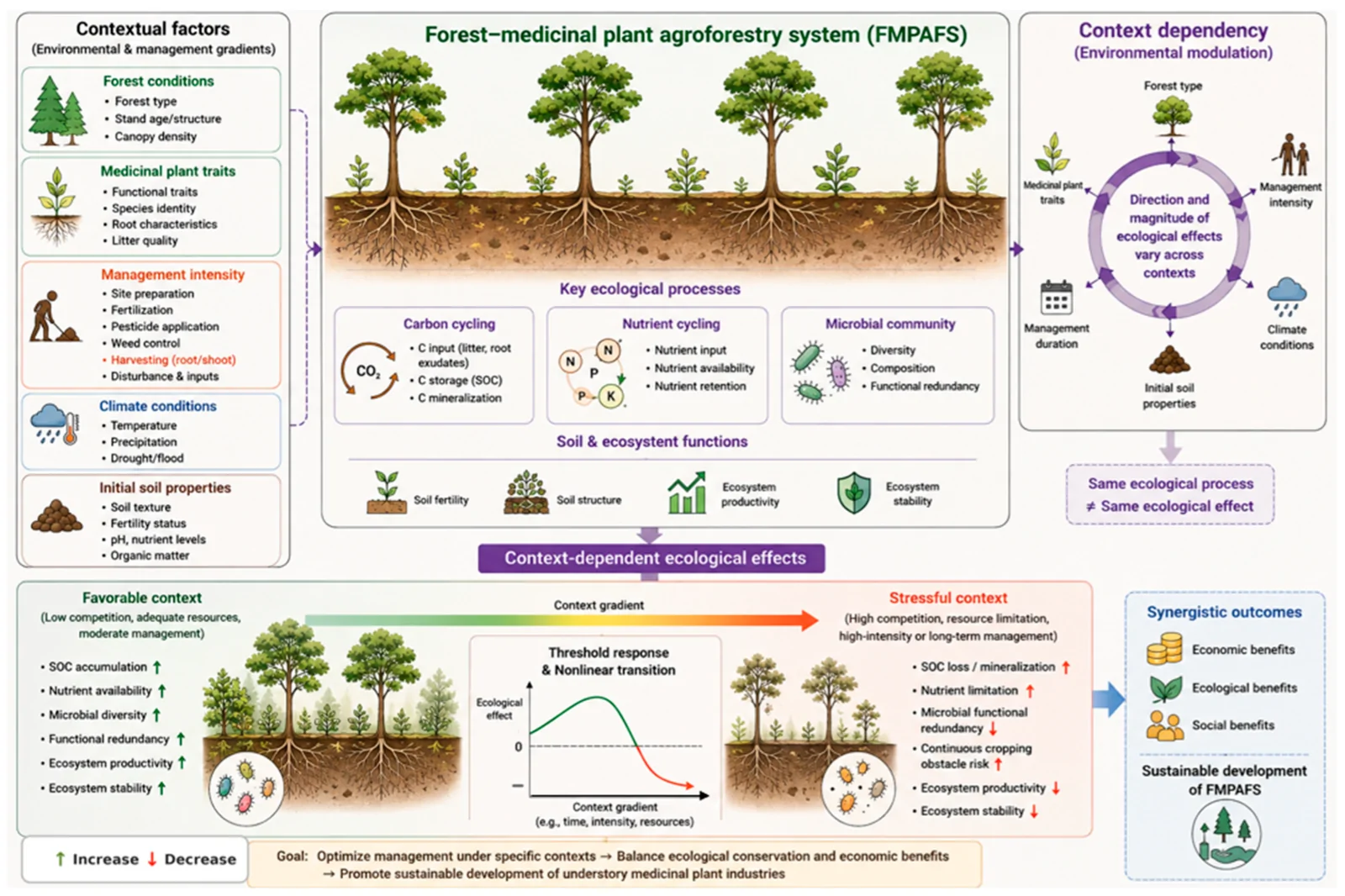
Conceptual framework illustrating the context-dependent ecological effects of FMPAFS. Contextual factors (**left**)—forest conditions, medicinal plant traits, management intensity, climate conditions, and initial soil properties—regulate key ecological processes (**center**), including carbon cycling, nutrient cycling, and microbial community assembly, which jointly determine soil and ecosystem functions. Because the direction and magnitude of these effects vary across contexts (**right**), the same ecological process does not necessarily produce the same ecological effect. Along the gradient from favorable (green) to stressful (red) contexts (bottom), ecological effects exhibit threshold responses and nonlinear transitions, and optimized management under specific contexts balances ecological conservation and economic benefits to promote the sustainable development of FMPAFS. Solid arrows indicate the direction of effects or flows; upward and downward arrows denote increases and decreases, respectively (see the legend); the green-to-red gradient represents the transition from favorable to stressful contexts; dashed boxes and dashed arrows indicate contextual modulation; in the threshold response plot, the y-axis denotes the ecological effect (positive above and negative below the zero line) and the x-axis denotes the context gradient (e.g., time, management intensity, and resource availability).

## Data Availability

No new data were created or analyzed in this study. Data sharing is not applicable to this article.
